# Do urinary tract infections affect morale among very old women?

**DOI:** 10.1186/1477-7525-8-73

**Published:** 2010-07-22

**Authors:** Irene Eriksson, Yngve Gustafson, Lisbeth Fagerström, Birgitta Olofsson

**Affiliations:** 1 Department of Community Medicine and Rehabilitation, Geriatric Medicine, Umeå University, Umeå, Sweden; 2 School of Life Sciences, University of Skövde, Skövde, Sweden; 3 Department of Health and Life Sciences, University of Buskerud, P.O Box 235, N-3603, Kongsberg, Norway; 4 Department of Nursing, Umeå University, Umeå, Sweden

## Abstract

**Background:**

Urinary tract infection (UTI) is among the most common bacterial infections in women of all ages but the incidence increases with older age. Despite the fact that UTI is a common problem it is still poorly investigated regarding its connection with experienced health and morale. The aim of this study was to explore the impact of a diagnosed, symptomatic urinary tract infection (UTI) with or without ongoing treatment on morale or subjective wellbeing among very old women.

**Methods:**

In a cross-sectional, population-based study, 504 women aged 85 years and older (range 84-104) were evaluated for ongoing UTI. Of these, 319 (63.3%), were able to answer the questions on the Philadelphia Geriatric Center Morale Scale (PGCMS) which was used to assess morale or subjective wellbeing.

**Results:**

In the present study sample of 319 women, 46 (14.4%) were diagnosed as having had a UTI with or without ongoing treatment when they were assessed. Women with UTI with or without ongoing treatment had significantly lower PGCMS scores (10.4 vs 11.9, p = 0.003) than those without UTI, indicating a significant impact on morale or subjective wellbeing among very old women. Depression (p < 0.001), UTI (p = 0.014) and constipation (p = 0.018) were the medical diagnoses significantly and independently associated with low morale in a multivariate regression model.

**Conclusions:**

As UTI seems to be independently associated with low morale or poor subjective wellbeing, there needs to be more focus on prevention, diagnosis and treatment of UTI in old women.

## Background

Urinary tract infection (UTI) is among the most common bacterial infections in women of all ages but the incidence increases with older age. Almost half of all women have suffered from at least one UTI sometime during their reproductive years and this increases to at least 60% in postmenopausal women [1-3]. Important risk factors are oestrogen deficiency, urinary retention, urinary incontinence, a prior history of UTI, sexual activity and diabetes [2-5]. UTI in older patients can be a complex problem in terms of approach to diagnosis, treatment and prevention because in older patients it frequently presents with a range of atypical symptoms such as delirium, gastrointestinal signs and falls [6-11]. Caregivers may not always understand the impact that an apparently trivial illness such as UTI has on the patient and successful treatment from a medical point of view may not always translate into enhanced quality of life [12].

Although uncomplicated UTI in women is considered to be a relatively benign and self-limiting condition, it has an effect on the quality of life and causes unnecessary suffering, for example in the form of weakness and a feeling of being ill [13,14]. Any illness, even if short-lived and not life-threatening, can have an important impact on the patient's daily activities, social functioning and wellbeing [15,16]. Acute cystitis, as well as a failure of the treatment, and adverse effects of antibiotics can reduce women's quality of life [17].

Quality of life is a multidimensional concept and could be difficult to define faced with the lack of a consensual definition. Subjective indicators, however, such as sense of wellbeing and satisfaction with life can describe the concept. The World Health Organization Quality of Life Group (WHOQOL) (1995) defined quality of life as the "individual's perception of their position in life in the context of the culture and value systems in which they live and with regard to their goals, expectations, standards and concerns" (p. 1403). Quality of life includes at a minimum physical, psychological and social dimensions. The physical dimension describes the individual's perception of their physical state, the psychological dimension the individual's perception of their cognitive and affective states and the social dimension describes the individual's perception of the interpersonal relationships and social roles in their life [18]. Various concepts, such as life satisfaction, subjective or psychological wellbeing and morale are used synonymously in the literature [19]. Morale, which we chose to use in this study, is defined by Lawton as a basic sense of satisfaction with oneself, a feeling that there is a place in the environment for oneself, and a certain acceptance of what cannot be changed [20]. Morale has been reported to be influenced by different medical conditions such as diabetes, stroke, depression, Parkinson's disease and heart failure [21-23]. Those with high morale are often active, sociable and optimistic in their attitudes but these attributes are not essential components of high morale [20]. Morale can be influenced by depression but it is not known whether low morale is a predictor of depression [22,23]. People can still have high morale even if their philosophy of life is pessimistic and if they are inactive and solitary [20]. Despite the fact that UTI is a common problem it is still poorly investigated regarding its connection with experienced health and morale. There is a lack of population-based studies in very old women with ongoing UTI and its association with morale. The purpose of this study was to explore whether a diagnosed symptomatic UTI with or without ongoing treatment had any impact on morale or subjective wellbeing among very old women.

## Methods

### Sample

**Figure 1:**
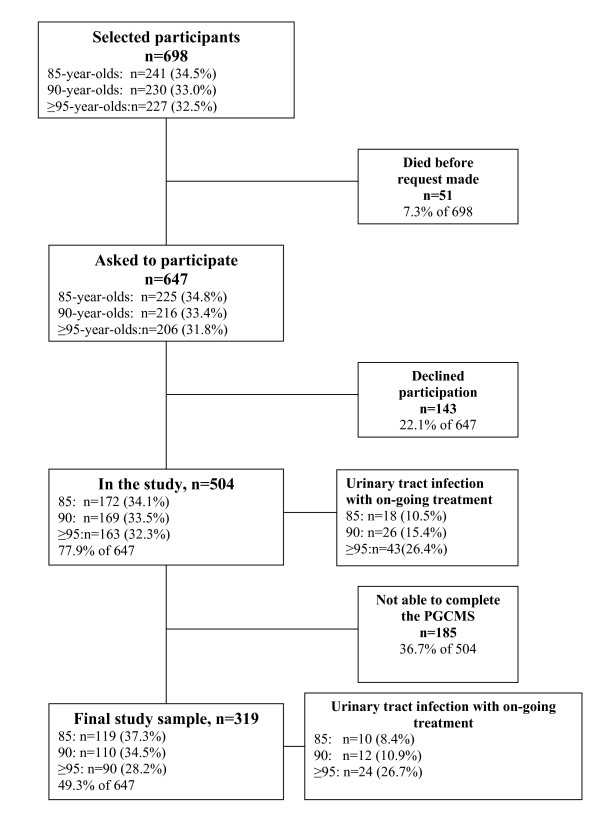
**Flow chart of the study population**.

This study is a part of the GErontological Regional DAtabase project (GERDA project), itself a continuation of the Umeå 85+ study that took place in the urban municipality of Umeå and five rural municipalities in the county of Västerbotten in Sweden 2005-2007 and in the municipalities of Vaasa and Mustasaari in Finland during 2005-2006 [24]. The subjects were selected from the population record, acquired from the Swedish and Finnish tax agencies respectively. A random sample, comprising half of the 85-year-olds, and the total population of 90-year-olds and ≥95-year-olds was selected for participation. Of the total sample of 698 women, 271 (38.8%) were from Finland and 427 (61.2%) from Sweden and 504 could be evaluated for UTI (Figure 1).

These 504 women comprised 85-year-olds (n = 172), 90-year-olds (n = 169) and ≥95-year-olds (n = 163). The Philadelphia Geriatric Center Morale Scale (PGCMS) was used to assess morale and 185 of the 504 women were unable to answer the questions or declined to receive home visits. They did not differ from the remaining sample regarding the prevalence of UTI but they were older and a larger proportion suffered from dementia. The final study sample consisted of 319 participants and comprised 85-year-olds (n = 119), 90-year-olds (n = 110) and ≥95-year-olds (n = 90).

## Procedure

The same procedure was used, as in the Umeå 85+ study, which has been described in detail earlier [24]. The investigator, who was a nurse, a physician, a physiotherapist or a medical student, made one or more home visits to those who gave their consent. Each home visit, including assessments and a structured interview, took approximately two hours to complete. Data were also collected from medical records, from hospitals and from the patient's general practitioner, and from caregivers and relatives.

### Social factors

The GERDA project includes information about social background variables such as living conditions and both participants living in their own homes and those living in institutions were included.

### Medical factors

Medical history and current health status as well as current drug use - both prescription and non-prescription drugs - were also included in the information. Reliable and well-known assessment scales were used. The Mini Mental State Examination (MMSE) was used to assess cognition in the participants. The scale has a maximum score of 30 with a score of 23 or less indicating impaired cognition [25]. The Geriatric Depression Scale-15 (GDS-15) was used to assess depressive symptoms. Scores of between five and nine indicate mild depression, and a score of ten or more indicates moderate to severe depression [26]

### Functional factors

Dependency in activities of daily living was assessed using the ADL Staircase (including the KATZ Index of ADL) which measures both Instrumental ADL and Personal ADL [27] and the Barthel ADL Index with a maximum score of 20 indicating independence in all personal ADL activities [28]. The participants' height and weight were assessed and Body Mass Index (BMI) calculated (kg/m^2^).

Based on all assessments, drug treatments and all documentation in medical records a specialist in geriatric medicine evaluated all data, in order to arrive at diagnoses, using the same criteria for all participants. Dementia and depression were diagnosed according to the DSM IV criteria, based on medical history, test results and medical record notes.

### Morale

Quality of life instruments for old people were reviewed by the British Geriatrics Society and the Royal College of Physicians of London. They recommend the use of the PGCMS for assessment of morale or subjective wellbeing among old people [29]. This study assessed morale using the 17-item British English version of the PGCMS, translated into Swedish [20,22,30]. The scores range from 0 to 17, where scores of 17-13 indicate high morale, 12-10 middle range and 9-0 low morale. The PGCMS is also comparatively easy to use in people with mild to moderate cognitive impairment since the questions only require yes/no answers [20,29]. In this study, the scale was interviewer administered.

### Definition of UTI

UTI was diagnosed if the person had a documented symptomatic UTI, with either short or long-term ongoing treatment with antibiotics, or symptoms and laboratory tests judged to indicate a UTI by the responsible physician or the assessor. Medical records from the general practitioner, from the hospitals in the catchment area or records from the caring institutions were also investigated to evaluate and validate the UTI diagnosis. The UTI diagnosis in the medical records was based on urinary tests in combination with symptoms that were judged to be associated with UTI by the responsible physician. In addition, the results from all urinary cultures registered at the regional bacteriological laboratory were reviewed. This means that the UTI diagnose was registered if the participants had symptoms and/or signs of UTI when they were assessed or had had a recent diagnosis of UTI.

### Data analysis

The χ^2 ^and Student's t-tests were used to analyze differences between groups and Pearson's correlation analyses were used for associations between continuous variables. A multivariate linear regression model was constructed, based on *a priori *hypotheses that morale could be influenced by medical conditions such as infections, diabetes, stroke, depression, Parkinson's disease and heart failure. Diagnoses that had a statistically significant association with low PGCMS scores were included in multivariate linear regression models to find the independent diagnoses associated with PGCMS scores. A p-value of < .05 was regarded as statistically significant. The Predictive Analytics Software (PASW) Statistics version 18 (SPSS Inc., Chicago, IL) was used for the calculations.

### Ethics

The study was approved by the Regional Ethical Review Board in Umeå (registration number 05-063M) and the Ethics Committee of Vaasa Central Hospital (registration number 05-87).

## Results

In the present study sample of 319 women, 46 (14.4%) were diagnosed as having had a UTI with or without ongoing treatment when they were assessed. Of the 46 women with a UTI, 10/119 (8.4%) were 85 years old, 12/110 (10.9%) were 90 years old and 24/90 (26.7%) were ≥95 years old. Almost two thirds of the 46 women had had two or more UTIs in the preceding year. The clinical characteristics of women who suffered from a UTI compared to those who did not are shown in Table 1. Of the 46 women with UTI, 31 had an ongoing treatment for UTI and in 15 cases, the assessor who made the home-visit, found documentation in the records and/or received information from the staff (responsible nurse) indicating UTI. In 12 of the 46 cases documentation of laboratory tests such as urinary cultures were found. The documentation included symptoms and laboratory tests. Participants diagnosed with depression, dementia, constipation, heart failure, stroke, impaired vision and UTI had significantly reduced morale according to the PGCMS, compared with those without these diagnoses (Table 2). Women with UTI had a mean score on the PGCMS of 10.4 ± 3.6 versus 11.9 ± 3.1 (p = 0.003) for those without UTI.

**Table 1:** Characteristics of women (n = 319) with and without urinary tract infection with ongoing treatment.

	UTI (n = 46)		NO UTI (n = 273)			THE TOTAL SAMPLE (n = 319)	
*Social factors*	n	%	n	%	p- value	n	%
Civil status (single) (n = 46/271)	45	97.8	246	90.8	0.107	291	91.8
Living alone	44	95.7	232	85.0	0.050	276	86.5
In institutional care	28	60.9	85	31.1	<0.001	113	35.4
*Medical factors*							
Constipation - current	23	50.0	102	37.4	0.104	125	39.2
Dementia	25	54.3	77	28.2	<0.001	102	32.0
Depression	22	47.8	100	36.6	0.148	122	38.2
Diabetes	8	17.4	41	15.0	0.680	49	15.4
Heart failure	23	50.0	82	30.0	0.008	105	32.9
Hip fractures	10	21.7	25	9.2	0.012	35	11.0
Indwelling catheter	5	10.9	1	0.4	<0.001	6	1.9
Impaired hearing (n = 45/270)	14	31.1	40	14.8	0.008	54	17.1
Impaired vision (n = 46/272)	9	29.6	45	16.5	0.354	54	17.0
Malignancies	6	13.0	19	7.0	0.156	25	7.8
Rheumatic disease	4	8.7	31	11.4	0.593	35	11.0
Stroke in the preceding five years	7	15.2	23	8.4	0.144	30	9.4
Urinary incontinence - current	20	43.5	78	28.6	0.043	98	30.7
*Functional factors *							
Eats independently according to KATZ (n = 46/272)	44	95.7	266	97.8	0.391	310	97.5
Goes outside independently according to KATZ (n = 45/271)	19	42.2	184	67.9	0.001	203	64.2
Independent in toileting according to KATZ (n = 46/272)	30	65.2	235	86.4	<0.001	265	83.3
Transfers independently (n = 46/272)	32	69.6	245	90.1	<0.001	277	87.1
	Mean ± SD		Mean ± SD			Mean ± SD	
Barthel's ADL index (n = 46/269)	13.7 ± 6.2		17.4 ± 4.1		<0.001	16.9 ± 4.7	
BMI (n = 42/264)	25.0 ± 3.9		25.7 ± 4.5		0.356	25.6 ± 4.4	
GDS (n = 45/271)	4.4 ± 2.7		3.5 ± 2.4		0.018	3.6 ± 2.5	
MMSE (n = 46/272)	19.7 ± 6.2		22.5 ± 5.4		0.002	22.0 ± 5.6	
Number of drugs	9.2 ± 3.9		6.6 ± 3.9		<0.001	7.0 ± 4.0	

**Table 2:** The total PGCMS scores for women (n = 319) with and without specific characteristics.

	Yes (n)	PGCMS Mean ± SD	No (n)	PGCMS Mean ± SD	p-value
*Social factors*					
Living alone	276	11.5 ± 3.2	43	12.6 ± 3.0	0.048
In institutional care	113	10.8 ± 3.5	206	12.1 ± 2.9	0.001
*Medical factors*					
Constipation	125	10.8 ± 3.2	194	12.2 ± 3.1	<0.001
Dementia	102	10.9 ± 3.2	217	12.0 ± 3.2	0.003
Depression	122	9.5 ± 3.2	197	13.0 ± 2.4	<0.001
Diabetes	49	11.9 ± 3.2	270	11.6 ± 3.2	0.538
Heart failure	105	11.1 ± 3.3	214	11.9 ± 3.1	0.028
Hip fractures	35	11.3 ± 3.4	284	11.7 ± 3.2	0.502
Indwelling catheter	6	8.2 ± 4.4	313	11.7 ± 3.2	0.007
Impaired hearing (n = 315)	54	11.1 ± 3.7	261	11.8 ± 3.1	0.182
Impaired vision (n = 318)	55	10.4 ± 3.0	263	11.9 ± 3.2	0.002
Malignancies	25	11.0 ± 3.5	294	11.7 ± 3.2	0.286
Rheumatic disease	35	11.4 ± 2.7	284	11.7 ± 3.3	0.576
Stroke in the preceding five years	30	9.7 ± 3.6	289	11.9 ± 3.1	<0.001
Urinary incontinence	98	11.2 ± 3.0	221	11.9 ± 3.3	0.066
Urinary tract infection - current	46	10.4 ± 3.6	273	11.9 ± 3.1	0.003
*Functional factors*					
Eats independently (n = 318)	310	11.7 ± 3.2	8	9.1 ± 4.0	0.024
Goes outside independently (n = 316)	203	12.2 ± 3.0	113	10.7 ± 3.2	<0.001
Independent in toileting (n = 318)	265	12.0 ± 3.1	53	10.1 ± 3.5	<0.001
Transfers independently (n = 318)	277	11.8 ± 3.2	41	10.4 ± 3.4	0.008

Participants living alone or in institutions had significantly reduced morale, according to the PGCMS. Lower PGCMS scores were also seen in participants who were dependent in eating, transfer and toileting, did not go outside, had an indwelling catheter and reduced vision (Table 2). The low PGCMS scores correlated significantly with high age, large number of drugs and low scores on Barthel's ADL index, GDS and MMSE (Table 3).

**Table 3:** Correlations between PGCMS and continuous predictor variables among the women (n = 319).

Predictor variables	m (sd)	Range	Correlation with PGCMS	p-value
Age	90.1 (4.6)	84-104	-.142	0.011
Barthel's ADL index	16.9 (4.7)	0-20	.235	<0.001
Body Mass Index	25.6 (4.4)	14.5-40	-.014	0.812
Geriatric Depression Scale	3.6 (2.5)	0-11	-.674	<0.001
Mini Mental State Examination	22.1 (5.6)	5-30	.205	<0.001
Number of drugs	7.0 (4.0)	0-19	-.211	<0.001

In the final multivariate linear regression model the diagnoses independently associated with low PGCMS scores were, depression (β = 3.31, p < 0.001), UTI (β = 1.07, p = 0.014) and constipation (β = 0.74, p = 0.018) and these three factors explained 31% of the variations of the PGCMS score (Table 4) while diagnoses such as urinary incontinence, heart failure, dementia and stroke did not qualify for the final multivariate linear regression model.

**Table 4:** Multivariate linear regression model of medical diagnoses associated with the total PGCMS scores (n = 318).

	β	95% CI	p-value
Depression	3.31	2.70-3.93	<0.001
Urinary tract infection with or without ongoing treatment	1.07	0.22-1.91	0.014
Constipation	0.74	0.13-1.36	0.018

p-value: < 0.001, R^2 ^= 0.309, Adjusted R^2 ^= 0.303

## Discussion

In the present study sample, 14% of very old women had a diagnosed UTI with or without ongoing treatment and the prevalence increased with age. UTI was associated with a significantly lower PGCMS score in this study and UTI, depression and constipation were the diagnoses independently associated with low morale in a multivariate regression model in old women. Diagnoses such as malignancies, rheumatic diseases, stroke, dementia, heart failure and diabetes were not significantly associated with low morale in the regression model. It was remarkable that although the women with UTI were receiving ongoing treatment at the time that they were assessed using the PGCMS, they nevertheless experienced low morale.

Old age is associated with reduced reserve capacity and in addition many old women suffer from multiple diseases. Very old women, as in this study, may have major responses to relatively minor insults such as infections and constipation. Thus, in a frail old woman a UTI might have a more serious impact on morale than in younger and healthier people. Another possible explanation might be that these women felt ill as a result of the medical treatment itself or because the treatment did not have the expected effect on the UTI. It has previously been shown that adverse effects of antibiotics as well as treatment failure can reduce quality of life [17]. Another explanation might be that these women have an enduring feeling of poor wellbeing over a long period of time, despite medical treatment of their UTI.

The association between UTI and morale among these old women in the present study is in line with previous findings from studies among younger women [14,16,31]. Women with UTI experience the symptoms in various ways but descriptions of the difficulty of enduring such symptoms as burning are common [31]. The symptoms are also described as a general feeling of being physically miserable as well as tired and irritable. The results indicate that UTI has a significant effect on morale despite the fact that the general opinion is that it is a "harmless" disease. A somewhat surprising finding in this study was that UTI with or without ongoing treatment - but not urinary incontinence - had a significant impact on morale in these old women. Especially since previous studies have found that, old women, suffering from urinary incontinence often have a reduced quality of life [32,33]. However, in the present study UTI in old women seems to be more important for morale than urinary incontinence. It is not unusual for UTI and urinary incontinence to have similar symptoms and sometimes incontinence itself is a symptom of a UTI. Thus it is sometimes possible to deal with urinary incontinence problems by treating the UTI. Nevertheless, it is important for the caregivers to be aware of both UTI and urinary incontinence, since both might have an impact on old women's morale.

As one might expect, in the present study depression was associated with low morale according to the PGCMS in the univariate analyses and also remained so in the final multivariate linear regression model. These findings are supported by previous studies [23,34] which have shown that depression is associated with a number of diagnoses, concomitant problems and disabilities in daily life. Depression among old women is common, it often remains undiagnosed and untreated, and influences their morale. In previous studies depression was found to be associated with institutional care, experienced loneliness and feeling unsafe [34,35]. In addition, depressed people more often suffered from constipation, dementia, osteoporosis, impaired vision, used a large number of medications, had lower scores on the MMSE and MNA and were older [34]. Although depression and low morale are closely connected they cannot be considered as synonymous because people with depression can have high morale and people with low morale are not always depressed [23]. The PGCMS and GDS scales measure different aspects of the person's well- or ill-being and using both scales is therefore worthwhile. High scores on the GDS are probably a better predictor of low morale than low PGCMS scores are of depression [23,36].

Women with constipation tend to have a poorer quality of life and low morale, which is supported by previous studies in younger old women and men [37,38]. Constipation seems to have a substantial impact on these women's activities of daily life and they experienced poorer health. It is common for there to be a difference between the patient's and physician's perceptions of the importance of the symptoms and how they affect the patient's daily life and morale [7,12,39]. The discrepancy between these perceptions could be an effect of poor patient-physician communication or differences in understanding of the illness [12,40].

Even if such conditions as UTI and constipation are in fact considered trivial illnesses and are not always regarded as important, they seem to have a significant impact on morale in old women [13,14]. These conditions are sometimes neglected and underdiagnosed, and underlying causes are often not investigated. UTIs in old women are frequently treated with antibiotics, but as prevention and treatment of underlying risk factors for UTI are often ignored recurrent UTI is common among these women. It is important for all care givers working with old women to pay attention to such common diagnoses as UTI and constipation since they are amenable to inexpensive and non invasive intervention. It is also important that they be aware of signs of low morale and use scales such as the PGCMS to identify such signs. Since low morale might be caused by underlying diseases, such as UTI and constipation, patients with low morale must be assessed for underlying causes.

The PGCMS is described as an appropriate instrument for measuring morale or subjective wellbeing among very old people [20,29]. The strength of this instrument lies in the scale, developed for use with older people, which is easily self- or interviewer- administered and also applicable to participants with mild and moderate cognitive impairment since the 17 questions can be answered with only yes or no [20,29,30]. The scoring of the PGCMS has an acceptable level of reliability, validity and a high internal consistency [20].

One limitation of the present study was that in the oldest age group, several women could not complete the PGCMS due to severe cognitive impairment. Another limitation was that no urinary tests or urine cultures were taken in conjunction with the home visits when the PGCMS was performed which makes it impossible to evaluate whether the participants with UTI with ongoing treatment had responded to treatment.

## Conclusions

UTI, depression and constipation are common among very old women and are associated with low morale or poor subjective wellbeing. More attention has to be given to very old women with UTI and UTI has to be prevented, detected and treated if these women are to have a good old age. Since there is a high incidence of UTI among old women combined with an ongoing increase in the older population, there is a great need for further research, such as intervention studies or how old women experience their health and life in general during an ongoing UTI.

## Conflict of interest statement

The authors declare that they have no competing interests.

## Authors' contributions

Study concept and design: YG and LF; Acquisition of data: BO and YG; analysis and interpretation of data: IE, YG, LF and BO; drafting of the manuscript: IE, YG, LF and BO; critical revision of the manuscript for important intellectual content: IE, YG, LF and BO; statistical analysis: IE and YG; obtaining funding: YG and LF; administrative, technical, and material support: YG

All authors have read and approved the final manuscript.

## References

[B1] Eriksson I Gustafson Y Fagerstrom L Olofsson B Prevalence and factors associated with urinary tract infections (UTIs) in very old women Arch Gerontol Geriatr 2010 50 2 132 135 10.1016/j.archger.2009.02.013 19349084

[B2] Foxman B Epidemiology of urinary tract infections: incidence, morbidity, and economic costs Am J Med 2002 113 Suppl 1A 5S 13S 10.1016/S0002-9343(02)01054-9 12113866

[B3] Harrington RD Hooton TM Urinary tract infection risk factors and gender J Gend Specif Med 2000 3 8 27 34 11253265

[B4] Molander U Arvidsson L Milsom I Sandberg T A longitudinal cohort study of elderly women with urinary tract infections Maturitas 2000 34 2 127 131 10.1016/S0378-5122(99)00102-4 10714907

[B5] Hu KK Boyko EJ Scholes D Normand E Chen CL Grafton J Fihn SD Risk factors for urinary tract infections in postmenopausal women Arch Intern Med 2004 164 9 989 993 10.1001/archinte.164.9.989 15136308

[B6] Laurila JV Laakkonen ML Tilvis RS Pitkala KH Predisposing and precipitating factors for delirium in a frail geriatric population J Psychosom Res 2008 65 3 249 254 10.1016/j.jpsychores.2008.05.026 18707947

[B7] Midthun S Paur R Bruce AW Midthun P Urinary tract infections in the elderly: a survey of physicians and nurses Geriatr Nurs (New York, NY 2005 26 4 245 251 10.1016/j.gerinurse.2005.06.011 16109298

[B8] Clayson D Wild D Doll H Keating K Gondek K Validation of a patient-administered questionnaire to measure the severity and bothersomeness of lower urinary tract symptoms in uncomplicated urinary tract infection (UTI): the UTI Symptom Assessment questionnaire BJU Int 2005 96 3 350 359 10.1111/j.1464-410X.2005.05630.x 16042729

[B9] Kallin K Jensen J Olsson LL Nyberg L Gustafson Y Why the elderly fall in residential care facilities, and suggested remedies J Fam Pract 2004 53 1 41 52 14709266

[B10] Manepalli J Grossberg GT Mueller C Prevalence of delirium and urinary tract infection in a psychogeriatric unit J Geriatr Psychiatry Neurol 1990 3 4 198 202 10.1177/089198879000300404 2073307

[B11] Shortliffe LM McCue JD Urinary tract infection at the age extremes: pediatrics and geriatrics Am J Med 2002 113 Suppl 1A 55S 66S 10.1016/S0002-9343(02)01060-4 12113872

[B12] Platt FW Keating KN Differences in physician and patient perceptions of uncomplicated UTI symptom severity: understanding the communication gap Int J Clin Pract 2007 61 2 303 308 10.1111/j.1742-1241.2006.01277.x 17263717

[B13] Foxman B Barlow R D'Arcy H Gillespie B Sobel JD Urinary tract infection: self-reported incidence and associated costs Ann Epidemiol 2000 10 8 509 515 10.1016/S1047-2797(00)00072-7 11118930

[B14] French L Urinary tract infection in women Adv Stud Med 2006 6 24 29

[B15] Ellis AK Verma S Quality of life in women with urinary tract infections: is benign disease a misnomer? J Am Board Fam Pract/Am Board Fam Pract 2000 13 6 392 397 10.3122/15572625-13-6-392 11117334

[B16] Wild DJ Clayson DJ Keating K Gondek K Validation of a patient-administered questionnaire to measure the activity impairment experienced by women with uncomplicated urinary tract infection: the Activity Impairment Assessment (AIA) Health Qual Life Outcomes 2005 3 42 10.1186/1477-7525-3-42 16022727 PMC1180845

[B17] Ernst EJ Ernst ME Hoehns JD Bergus GR Women's quality of life is decreased by acute cystitis and antibiotic adverse effects associated with treatment Health Qual Life Outcomes 2005 3 45 10.1186/1477-7525-3-45 16048650 PMC1183236

[B18] The World Health Organization Quality of Life assessment (WHOQOL): position paper from the World Health Organization Soc Sci Med (1982) 1995 41 10 1403 1409 10.1016/0277-9536(95)00112-k 8560308

[B19] Ranzijn R Luszcz M Measurement of subjective quality of life of elders Int J Aging Hum Dev 2000 50 4 263 278 10.2190/4B0W-AMGU-2NDX-CYUQ 11087107

[B20] Lawton M Kent D, Kastenbaum R, Sherwood S The dimensions of morale Research Planning and Action for the Elderly: The Power and Potential of Social Science 1972 New York: Behavioral Publications 144 65

[B21] Benito-Leon J Louis ED Bermejo-Pareja F Population-based case-control study of morale in Parkinson's disease Eur J Neurol 2009 16 3 330 336 10.1111/j.1468-1331.2008.02428.x 19170745

[B22] Lofgren B Gustafson Y Nyberg L Psychological well-being 3 years after severe stroke Stroke 1999 30 3 567 572 10066853 10.1161/01.str.30.3.567

[B23] von Heideken Wagert P Ronnmark B Rosendahl E Lundin-Olsson L Gustavsson JM Nygren B Lundman B Norberg A Gustafson Y Morale in the oldest old: the Umea 85+ study Age Ageing 2005 34 3 249 255 10.1093/ageing/afi044 15784647

[B24] von Heideken Wagert P Gustavsson JM Lundin-Olsson L Kallin K Nygren B Lundman B Norberg A Gustafson Y Health status in the oldest old. Age and sex differences in the Umea 85+ Study Aging Clin Exp Res 2006 18 2 116 126 16702780 10.1007/BF03327426

[B25] Folstein MF Folstein SE McHugh PR "Mini-mental state". A practical method for grading the cognitive state of patients for the clinician J Psychiatr Res 1975 12 3 189 198 10.1016/0022-3956(75)90026-6 1202204

[B26] Sheikh J Yesavage J Geriatric Depression Scale(GDS): recent evidence and development of a shorter version Clin Gerontol 1986 5 165 172 10.1300/J018v05n01_09

[B27] Katz S Ford AB Moskowitz RW Jackson BA Jaffe MW Studies of Illness in the Aged. the Index of Adl: a Standardized Measure of Biological and Psychosocial Function Jama 1963 185 914 919 14044222 10.1001/jama.1963.03060120024016

[B28] Mahoney F Barthel D Functional Evaluation: the Barthel Index Maryland State Med J 1965 14 62 14258950

[B29] Dall J Hopkins A Standardised assessment scales for elderly people 1992 London; UK: The Royal College of Physicians of London and the British Geriatrics Society 10.1136/jech.46.6.628 PMC1059683 1494081

[B30] Lawton MP The Philadelphia Geriatric Center Morale Scale: a revision J Gerontol 1975 30 1 85 89 1109399 10.1093/geronj/30.1.85

[B31] Malterud K Baerheim A Peeing barbed wire. Symptom experiences in women with lower urinary tract infection Scand J Prim Health Care 1999 17 1 49 53 10.1080/028134399750002908 10229994

[B32] Teunissen D Van Den Bosch W Van Weel C Lagro-Janssen T "It can always happen": the impact of urinary incontinence on elderly men and women Scand J Prim Health Care 2006 24 3 166 173 10.1080/02813430600739371 16923626

[B33] Temml C Wehrberger C Riedl C Ponholzer A Marszalek M Madersbacher S Prevalence and correlates for interstitial cystitis symptoms in women participating in a health screening project Eur Urol 2007 51 3 803 808 discussion 809 10.1016/j.eururo.2006.08.028 16979286

[B34] Bergdahl E Gustavsson JM Kallin K von Heideken Wagert P Lundman B Bucht G Gustafson Y Depression among the oldest old: the Umea 85+ study Int Psychogeriatr 2005 17 4 557 575 10.1017/S1041610205002267 16185377

[B35] Wilson K Mottram P Sixsmith A Depressive symptoms in the very old living alone: prevalence, incidence and risk factors Int J Geriatr Psychiatry 2007 22 4 361 366 10.1002/gps.1682 17044137

[B36] Coleman PG Philp I Mullee MA Does the use of the Geriatric Depression Scale make redundant the need for separate measures of well-being on geriatrics wards? Age Ageing 1995 24 5 416 420 10.1093/ageing/24.5.416 8669346

[B37] Glia A Lindberg G Quality of life in patients with different types of functional constipation Scand J Gastroenterol 1997 32 11 1083 1089 10.3109/00365529709002985 9399387

[B38] Wald A Scarpignato C Kamm MA Mueller-Lissner S Helfrich I Schuijt C Bubeck J Limoni C Petrini O The burden of constipation on quality of life: results of a multinational survey Aliment Pharmacol Ther 2007 26 2 227 236 10.1111/j.1365-2036.2007.03376.x 17593068

[B39] Simren M Svedlund J Posserud I Bjornsson ES Abrahamsson H Health-related quality of life in patients attending a gastroenterology outpatient clinic: functional disorders versus organic diseases Clin Gastroenterol Hepatol 2006 4 2 187 195 10.1016/S1542-3565(05)00981-X 16469679

[B40] Muntlin A Gunningberg L Carlsson M Patients' perceptions of quality of care at an emergency department and identification of areas for quality improvement J Clin Nurs 2006 15 8 1045 1056 10.1111/j.1365-2702.2006.01368.x 16879549

